# Determinants of care-seeking behaviour for fever, acute respiratory infection and diarrhoea among children under five in Nigeria

**DOI:** 10.1371/journal.pone.0273901

**Published:** 2022-09-15

**Authors:** Ifeoluwa Gbemisola Adeoti, Francesca L. Cavallaro

**Affiliations:** 1 Children Specialist Hospital, Ilorin, Kwara State, Nigeria; 2 Institute of Child Health, University College London, London, United Kingdom; University of Salamanca, SPAIN

## Abstract

**Background:**

Despite available, inexpensive and effective treatments, malaria, diarrhoea, and pneumonia still contribute the majority of the global burden of childhood morbidity and mortality. Nigeria has the highest absolute numbers of child deaths worldwide. Appropriate care-seeking is important for prompt diagnosis, appropriate and timely treatment, and prevention of complications. The objective of this cross-sectional study was to examine the prevalence of and factors associated with appropriate care-seeking for childhood illnesses.

**Methods:**

We used the most recent Nigeria Demographic and Health Survey (2018) to assess the prevalence of appropriate care-seeking among mothers of children under five with symptoms of common childhood illnesses. For diarrhoea, we defined appropriate care-seeking as seeking care from a formal health provider. For fever and acute respiratory infection (ARI), appropriate care-seeking was defined as seeking care from a formal provider the day of or after symptom onset. Multivariate logistic regression was carried out to assess factors associated with optimal care-seeking for each illness.

**Results:**

At least 25% of parents did not seek any care for children with fever or ARI; this figure was over one third for diarrhoea. Only 15% and 13% of caregivers showed appropriate care-seeking for their children with fever and ARI respectively, and 27% of mothers sought care from a formal provider for diarrhoea. Predictors of appropriate care-seeking varied according to childhood illness. Previous facility delivery was the only risk factor associated with increased odds of appropriate care-seeking for all three illnesses; other risk factors varied between illnesses.

**Conclusion:**

Overall, care-seeking for childhood illnesses was suboptimal among caregivers in Nigeria. Interventions to increase caregivers’ awareness of the importance of appropriate care-seeking are needed alongside quality of care interventions that reinforce people’s trust in formal health facilities, to improve timely care-seeking and ultimately reduce the high burden of child deaths in Nigeria.

## Introduction

The global under-five mortality rate has halved between 1990 and 2015, from 90 to 43 deaths per 1000 live births [1]. However, an unacceptably high number of children under five continue to die each year, with a large percentage of deaths due to pneumonia (15%), diarrhoea (8%) and malaria (5%) [1].

Nigeria recently overtook India as the country contributing the largest number of child deaths globally (844,000 deaths per year) [2]. Although under-five mortality decreased from 211 per 1,000 live births in 1990 to 114 in 2020 [2], Nigeria failed to achieve the Millennium Development Goal 4 to reduce childhood mortality by two-thirds. Important geographical and socio-economic variation in child mortality exists [3], however malaria, diarrhoea and pneumonia remain the main causes of post-neonatal child mortality in Nigeria [4].

Malaria, diarrhoea and pneumonia have inexpensive and effective treatments, including antibiotics, antimalarials, oral rehydration solution (ORS) and Zinc [5–7]. However, despite the availability of these treatments, many children receive care too late or receive the wrong treatment, because caregivers do not seek care, seek care late or from informal sources of care. Appropriate care-seeking–or the seeking of medical intervention for illness from trained medical personnel, in formal health facilities, and in a timely manner [8, 9]–allows for prompt and correct diagnosis, adequate management and prevention of complications.

A review of care-seeking behaviour in developing countries found that the majority of caregivers (73%) sought care for childhood illnesses, but only 45% sought care from formal providers [10]. In Nigeria, only 31% of under-five children with fever were reportedly taken to a formal health facility for treatment in 2013 [11]. Early care-seeking is also rare, with only 22% of community cases of malaria in South-Eastern Nigeria [12] and 57% of children hospitalised for fever in North-Central [11] having sought treatment within 24 hours. Improving parents’ care-seeking behaviour (thereby reducing the first delay in the three delays framework) [13, 14] can help prevent complications and child deaths; it is therefore imperative to better understand factors that affect appropriate care-seeking behaviour [15]. Although suboptimal care-seeking practices have been widely documented in Nigeria [11, 12, 16, 17], studies have usually focused either on timeliness of care-seeking or place of care.

The objective of our study was to examine the prevalence and determinants of appropriate care-seeking behaviour–including both timely care and from a formal provider–for children under 5 years in Nigeria presenting with symptoms of three common childhood illnesses: fever (as a common presenting symptom of malaria), diarrhoea, and acute respiratory infection (ARI; as a common presenting symptom of pneumonia).

## Materials and methods

### Setting

Nigeria is the most populous country in Africa, with 206 million inhabitants in 36 states and a very young population structure (43% of the population is aged below 15 years) [18]. In addition to accounting for the largest number of child deaths globally, Nigeria contributes the largest percentage of malaria cases (27%) and deaths (32%) worldwide, the majority of which are among children under five [19].

### Study design

This study was a secondary analysis of the 2018 Nigeria Demographic and Health Survey (DHS) [3]. The DHS are cross-sectional, nationally representative household surveys, widely used for generating population and health indicators in low- and middle-income countries. The sampling design was based on a two-stage cluster strategy (households selected within Enumeration Areas of the Population and Housing Census of the Federal Republic of Nigeria). Our analysis used information collected in the Woman’s and Household questionnaires. Respondents did not receive a financial incentive for participating; the response rate among eligible women was 99%.

**Fig 1:**
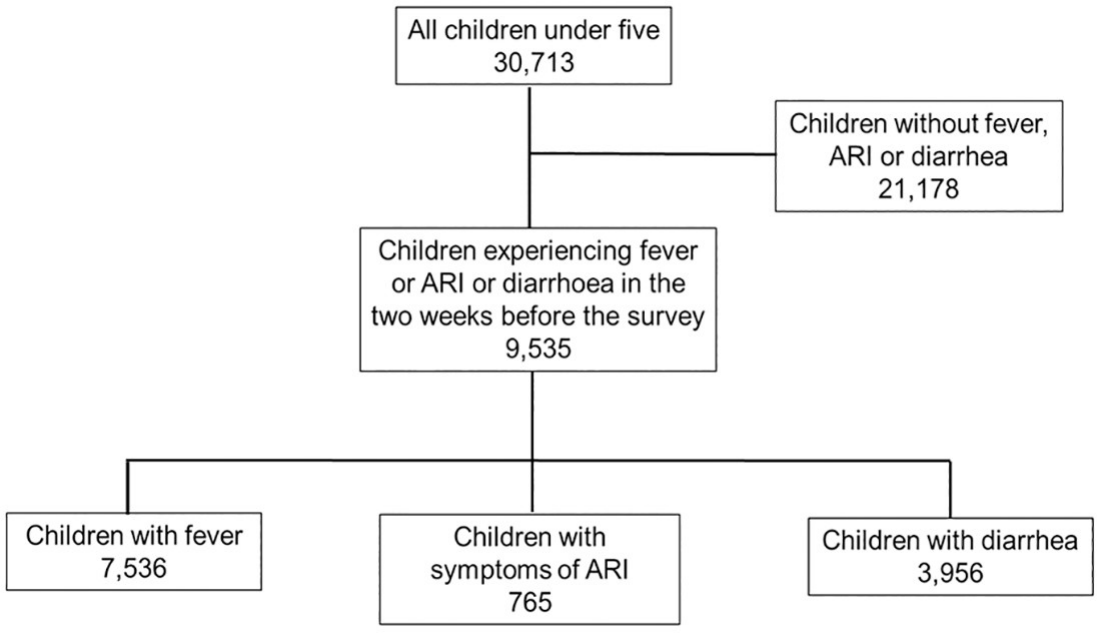
Summary of study sample. Some children had more than one symptom, so the total number of children experiencing the three illnesses is not equal to the sum of children experiencing each individual symptom.

Mothers aged 15–49 were asked whether each of their children under five experienced fever, diarrhoea, or symptoms of ARI (defined as chesty cough with short or rapid breathing) in the two weeks before the survey. If symptoms were reported, for all three symptoms mothers were asked whether they had sought care for the child and if so, from which provider; for children with fever and symptoms of ARI, mothers were additionally asked how many days after the onset of symptoms they sought care. A total of 30,713 children under five of women respondents were included in the sample (Fig 1). All 9,535 children with reported fever, diarrhoea or symptoms of ARI during the two weeks preceding the survey were included in the analysis (some children experienced more than one symptom, and were included in the relevant analyses for each symptom experienced).

### Definition of appropriate care-seeking for childhood illnesses

Fever and ARI both should be evaluated by a health care professional in all cases. For these two diseases, we therefore defined appropriate care-seeking as seeking care from a formal health provider on the same day or day after the onset of symptoms. However, mild and moderate dehydration from diarrhoea can be managed at home with ORS and zinc, and only severe dehydration or vomiting require intravenous fluid replacement at a formal health facility. Therefore, timing of care-seeking for diarrhoea is not collected in the DHS, and we only examined care-seeking from formal providers for children with diarrhoea.

We considered formal health providers to include hospitals (public and private), primary health care (PHC) facilities of any sector (including mobile clinics), and healthcare workers (private doctors, fieldworkers, community health worker). Pharmacies, shops, markets, traditional practitioners, itinerant drug sellers and other undisclosed sources were considered informal sources of care, as they are not authorised or trained to prescribe medicine, nor are they part of formal healthcare referral pathways.

### Determinants of care-seeking behaviour

**Fig 2:**
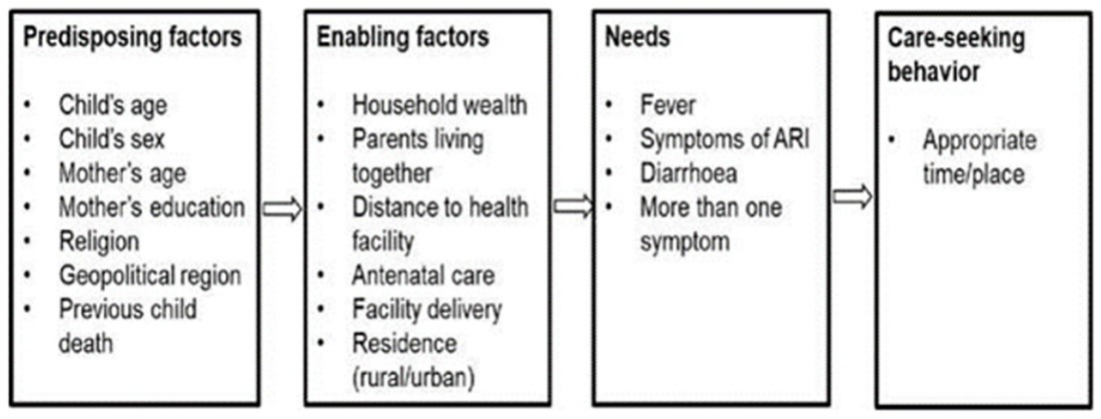
Predictors of care-seeking behaviour, adapted from Andersen and Davidson [21].

We adapted Andersen and Davidson’s behavioural model of health services use [11, 20, 21] for care-seeking behaviour for childhood illnesses based on information available in the dataset and a literature review (Fig 2). Predisposing factors included child age and sex, mother’s age, parental education, religion, geopolitical zone, and previous child death (hypothesised to encourage caregivers to seek care earlier). Enabling factors affecting ability to access care included household wealth quintile, urban/rural residency, mother’s marital status, antenatal care and facility delivery for the most recent live birth, and whether distance to health facility was reported a problem in accessing care. We also examined presence of multiple symptoms as a determinant of care-seeking behaviour.

### Statistical analysis

The children’s recode dataset for the 2018 Nigeria DHS dataset was analysed using Stata version 15.0. We described the sociodemographic characteristics of all children under five included in this study, and the prevalence of the three common childhood illnesses (fever, ARI and diarrhoea) according to these characteristics.

Among children with reported fever or ARI in the past two weeks, we calculated the percentage for whom any care was sought, care was sought at the appropriate time, care was sought from a formal provider, and care was sought at appropriate time and place (appropriate care-seeking). For diarrhoea, we calculated the percentage of children for whom any care was sought, and care was sought from a formal provider. The health providers from whom care was sought were also described for each illness.

We used logistic regression to calculate crude and adjusted odds ratios (with 95% confidence intervals) for the association between potential determinants and appropriate care-seeking (formal provider for diarrhoea), separately for each illness to compare determinants of care-seeking across the three common childhood illnesses. Variables with a p-value less than 0.15 in bivariate associations were included in the multivariable model, to estimate the independent effect of risk factors (adjusting for other risk factors associated with the outcome). We combined missing values with other categories in some models, to obtain more stable estimates.

The Stata svyset command was used to account for the multi-stage sampling design (including sampling weights, clustering and stratification) in all analyses.

### Ethics

The Institutional Review Board of ICF International and the Nigeria Health Research Ethics Committee of the Federal Ministry of Health granted ethical approval for the 2018 Nigeria DHS. Informed consent was obtained from participants, and privacy and confidentiality was maintained during data collection. This study being a secondary data analysis of de-identified data, secondary ethical approval was not required by the Institute of Child Health, University College London.

## Results

Of the 30,713 children under 5 years included in this study, 7,536 (24.3%) had fever, 3,956 (12.8%) had diarrhoea and 765 (2.3%) had symptoms of ARI in the two weeks preceding the survey, based on mothers’ report (Table 1). The prevalence of all three childhood illnesses was higher among children aged 12–23 months, children living in rural areas and in the North-East region, children whose father had no education, whose mother was less than 20 years old, and from the poorest households. The prevalence of childhood illnesses was also higher among women reporting that distance was a big problem in accessing health care and those who had experienced a previous child death. Prevalence of fever, ARI and diarrhoea was similar by child sex and maternal marital status.

**Table 1:** Socioeconomic and demographic characteristics of children under 5 and prevalence of fever, diarrhoea and symptoms of acute respiratory infection (ARI) during the two weeks preceding the survey.

Characteristics	All children under 5years (%)	Prevalence of fever (%)	Prevalence of symptoms of ARI (%)	Prevalence of diarrhoea (%)
**TOTAL**	**30,713**	**24.3 (N = 7,536)**	**2.3 (N = 765)**	**12.8 (N = 3,956)**
**Child’s age (months)**				
0–11	6,399 (20.8)	22.0	2.9	14.8
12–23	6,059 (19.9)	29.8	3.1	20.3
24–35	5,834 (18.9)	26.5	2.1	13.9
36–47	6,168 (20.0)	23.1	1.7	8.7
48–59	6,253 (20.4)	20.3	1.9	6.7
**Child’s sex**				
Male	15,537 (50.8)	23.7	2.3	12.8
Female	15,176 (49.3)	24.9	2.4	12.9
**Mother’s Age**				
<20	1,286 (4.2)	30.6	2.8	20.1
20–34	21,583 (70.6)	23.8	2.4	12.8
Above 35	7,844 (25.2)	24.5	2.2	11.6
**Mother’s education**				
No education	13,527 (44.9)	28.8	2.8	16.3
Primary	4,776 (15.0)	24.7	3.0	13.1
Secondary and above	12,410 (40.1)	19.0	1.6	8.9
**Father’s education**				
No education	10,079 (33.8)	29.0	2.8	16.1
Primary	4,186 (13.1)	25.1	2.7	11.9
Secondary and above	14,528 (47.1)	20.6	1.9	10.6
Missing	1,920 (6.1)	24.8	2.3	13.7
**Mother’s marital status**				
Single	1,532 (4.6)	23.3	2.9	12.2
In union	29,181 (95.4)	24.3	2.3	12.9
**Mother’s religion**				
Catholic	2,744 (8.7)	18.6	0.9	7.4
Other Christian	9,594 (28.4)	19.0	2.0	7.3
Islam	18,113 (62.3)	27.5	2.7	16.2
Traditionalist	108 (0.4)	24.0	0.6	5.8
Other	154 (0.2)	6.2	0	1.8
**Household wealth quintile**				
Poorest	7,081 (21.5)	32.6	3.8	18.7
Poorer	6,839 (22.1)	28.4	2.7	15.5
Middle	6,509 (20.6)	23.8	2.1	12.2
Richer	5,747 (18.8)	19.5	1.7	9.8
Richest	4,537 (17.0)	14.2	1.2	6.2
**Residence**				
Urban	10,851 (39.6)	18.6	1.7	9.6
Rural	19,862 (60.5)	27.9	2.8	14.9
**Region**				
North East	6,481 (18.1)	35.2	7.8	24.7
North Central	5,403 (13.8)	17.8	0.9	11.5
North West	8,934 (35.2)	28.0	1.1	13.9
South East	3,545 (10.4)	20.1	1.4	6.1
South South	3,021 (9.0)	25.6	2.3	6.1
South West	3,329 (13.5)	8.7	0.5	5.2
**Antenatal care visits (mother’s most recent live birth)**				
None	7,579 (24.4)	26.3	2.3	13.9
At least one	22,680 (74.0)	23.9	2.4	12.6
Missing	454 (1.6)	10.5	0.9	6.0
**Facility delivery (mother’s most recent live birth)**				
Yes	12,373 (40.0)	19.0	1.7	9.3
No	18,340 (60.0)	27.8	2.8	15.2
**Distance to health facility as barrier to accessing care (mother’s self-report)**				
Not important barrier	21,575 (72.1)	23.6	2.1	12.3
Important barrier	9,138 (28.0)	25.9	3.1	14.1
**Mother experienced previous child death**				
Yes	8,796 (28.8)	31.0	2.7	16.9
No	21,917 (71.2)	21.5	2.2	11.2
**Prevalence of at least one other symptom**^a^ **(N, %)**				
Yes	-	32.3%	76.4%	55.4%

^a^ARI and/or diarrhoea for fever, fever and/or diarrhoea for ARI, and fever and/or ARI for diarrhoea

Note: prevalence estimates exclude 112 children with missing data for fever and 110 children with missing data for diarrhoea.

### Care-seeking at appropriate place and time

Care-seeking behaviour was similar for children with fever and ARI. The majority of caregivers sought care for their children with fever (73%) and ARI (76%), however only 38% and 29%, respectively, sought help on the same day or day after the onset of illness (Table 2). Less than half sought care in formal providers. Overall, only 15% of children with fever and 13% of children with ARI symptoms had care sought appropriately.

**Table 2:** Care-seeking behaviour among children with fever, ARI and diarrhoea.

Childhood illness	Prevalence of illnessin last 2 weeks (% among all children)	N with illness	Any care sought (% among ill children)	Care sought from formal provider (% among ill children)^a^	Care sought at appropriate time^b^ (% among ill children)	Care sought at appropriate place and time^b^ (% among ill children)
**Fever**	24.3	7,536	72.8	30.9	38.2	14.9
**ARI**	2.3	765	75.6	39.7	28.9	13.3
**Diarrhoea**	12.8	3,956	64.9	26.7	N/A	N/A

^a^Formal provider: hospitals (public and private), primary health care (PHC) facilities (public and private health centres, posts, mobile clinics, private doctors, fieldworkers, community health workers, and NGO and other public sector facilities that are government-led).

^b^Appropriate time: seeking care on the same day or day after the onset of symptoms

ARI–Acute Respiratory Infection

In contrast, care was sought for fewer children with diarrhoea (65%), and only (27%) were taken to a formal provider. 24% of children with diarrhoea were treated with ORS and zinc at home.

**Fig 3:**
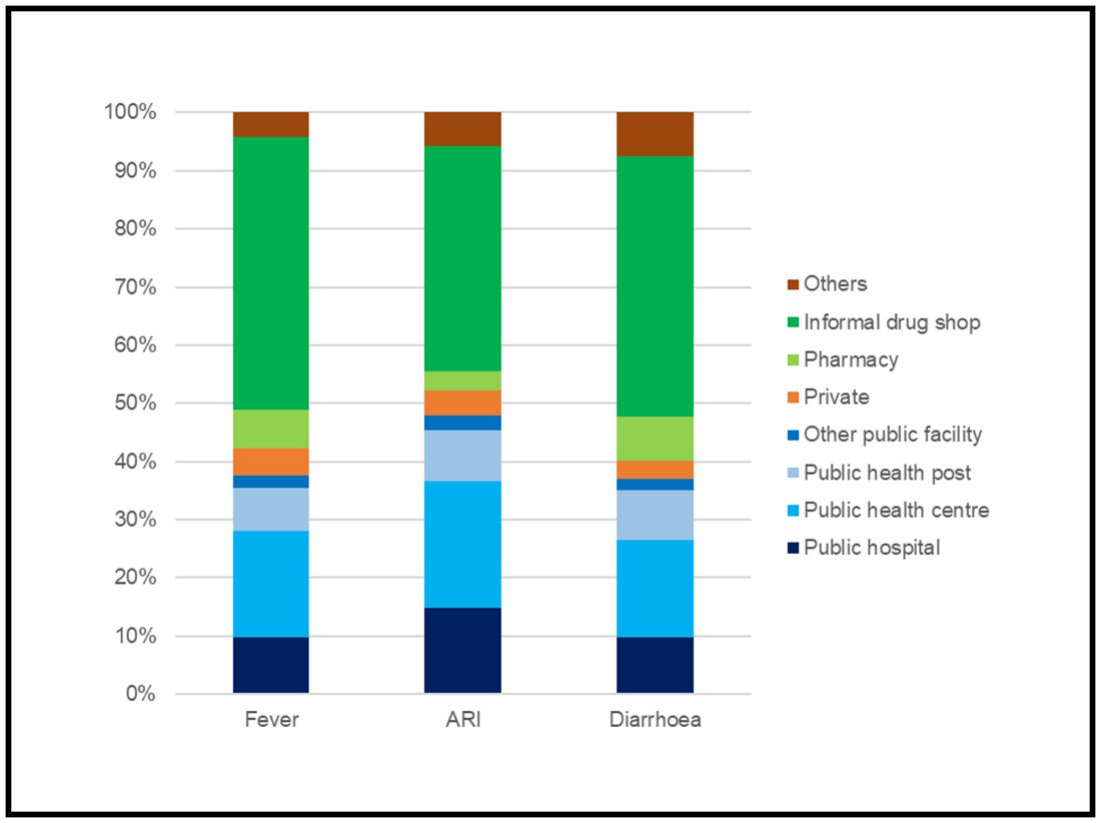
Sources where care was sought by caregivers of children under five with fever, ARI symptoms and diarrhoea in Nigeria (2018).

The most common place where care was sought was informal drug shops (without a trained pharmacist), accounting for 39%-47% across all three illnesses (Fig 3). Public health centres were the second most common source of care (however, public and private health facilities combined were the most common care-seeking place for children with ARI symptoms). Between 4–8% of caregivers sought care from “other” informal sources including traditional practitioners, shops, markets and itinerant drug sellers.

### Determinants of appropriate care-seeking

Among children with fever, there was strong evidence that paternal education, religion, wealth and region of residence were associated with appropriate care-seeking, after adjusting for other risk factors (Table 3). Children of mothers residing in the North-West region (Adjusted Odds Ratio (AOR) = 1.70 [95% CI: 1.33–2.15]) were more likely to receive appropriate care-seeking for fever, compared to those living in the North-East. The odds of appropriate care-seeking increased with father’s education (AOR = 1.49 [1.19–1.86] for secondary/higher education, compared with none) and household wealth (AOR = 1.76 [1.15–2.46] for the richest quintile compared to the poorest). Children of Muslim women had higher odds of appropriate care-seeking than Christians (AOR = 1.68 [1.25–2.31]). Children whose mothers did not report distance to health facility as a barrier in accessing care, received antenatal care for their most recent live birth, gave birth in a health facility and had not experienced previous child death had between 1.21–1.59 higher odds of appropriate care-seeking. Maternal age, education and residence were not associated with appropriate care-seeking in the multivariable model.

**Table 3:** Logistic regression analysis of factors associated with appropriate care-seeking behaviour for children under five with fever (N = 7,536).

Characteristics	N children	Appropriate care-seeking (%)	Crude OR (95% CI)	p-value	Adjusted OR (95% CI)	p-value
**Child’s age (months)**				0.785		
0–11	1,405	14.2	1.00		-	
12–23	1,857	15.7	1.13 (0.89–1.43)		-	
24–35	1,559	15.2	1.09 (0.85–1.38)		-	
36–47	1,426	14.5	1.02 (0.80–1.31)		-	
48–59	1,289	14.3	1.01 (0.79–1.29)		-	
**Child’s sex**				0.893		
Male	3,782	14.9	1.00		-	
Female	3,754	14.8	0.99 (0.86–1.14)		-	
**At least one additional symptom (ARI or diarrhoea)**				0.295		
Yes	2,441	14.1	0.92 (0.78–1.08)			
No	5,095	15.2	1		1	
**Mother’s Age**				0.094		0.352
<20	370	16.3	1		1.00	
20–34	5,248	15.4	0.93 (0.66–1.32)		0.89 (0.60–1.25)	
Above 35	1,918	13.1	0.54 (0.54–1.11)		0.78 (0.53–1.15)	
**Mother’s education**				<0.001		0.113
No education	3,950	11.7	1.00		1.00	
Primary	1,208	15.7	1.41 (1.13–1.76)		1.23 (0.96–1.57)	
Secondary and above	2,378	19.8	1.86 (1.53–2.26)		1.30 (0.99–1.71)	
**Father’s education**				<0.001		0.003
No education	2,977	10.6	1.00		1.00	
Primary	1,056	12.3	1.18 (0.91–1.52)		1.07 (0.81–1.42)	
Secondary and above	3,041	20.1	2.11 (1.73–2.57)		1.54 (1.20–1.98)	
Missing	462	14.7	1.44 (1.01–2.05)		1.27 (0.87–1.85)	
**Mother’s marital status**				0.898		
Single	348	15.2	1.00		-	
In union	7,188	14.9	0.98 (0.68–1.41)		-	
**Mother’s religion**				0.102		<0.001
Christianity/others	2,420	13.5	1.00		1.00	
Islam	5,116	15.5	1.18 (0.97–1.43)		1.76 (1.29–2.39)	
**Household wealth quintile**				<0.001		0.019
Poorest	2,300	10.3	1.00		1.00	
Poorer	1,868	13.2	1.33 (1.05–1.67)		1.06 (0.83–1.35)	
Middle	1,563	15.4	1.59 (1.24–2.03)		1.02 (0.76–1.36)	
Richer	1,139	17.9	1.90 (1.47–2.45)		1.09 (0.78–1.52)	
Richest	666	26.9	3.20 (2.37–4.34)		1.68 (1.15–2.46)	
**Residence**				<0.001		0.513
Urban	2,146	19.2	1.00		1.00	
Rural	5,390	13.0	0.63 (0.52–0.76)		1.08 (0.86–1.34)	
**Geopolitical zone**				<0.001		<0.001
North East	2,270	11.2	1		1	
North Central	949	12.7	1.15 (0.85–1.55)		1.03(0.76–1.40)	
North West	2,554	17.2	1.65 (1.28–2.11)		1.70 (1.33–2.15)	
South East	753	14.2	1.31 (0.95–1.82)		1.13 (0.73–1.75)	
South South	688	11.8	1.05 (0.71–1.57)		0.79(0.48–1.30)	
South West	322	26.4	2.84(1.87–4.33)		1.47 (0.95–2.28)	
**Antenatal care visits (mother’s most recent live birth)**				<0.001		<0.001
None	1,948	8.6	1		1	
At least one	5,520	16.9	2.16 (1.72–2.72)		1.59 (1.24–2.03)	
Missing	68	42.7	7.92 (3.41–18.39)		5.43 (2.52–11.71)	
**Facility delivery (mother’s most recent live birth)**				<0.001		0.002
No	5,126	12.4	1		1	
Yes	2,410	20.3	1.81 (1.51–2.17)		1.39 (1.13–1.69)	
**Distance to health facility as barrier to accessing care (mother’s self-report)**				<0.001		0.041
Important barrier	2,420	10.0	1		1	
Not important barrier	5,116	16.9	1.84 (1.51–2.25)		1.25 (1.01–1.54)	
**Mother experienced previous child death**				<0.001		0.024
No	4,805	16.4	1		1	
Yes	2,731	12.2	0.71 (0.60–0.83)		0.82 (0.69–0.97)	

Among children with symptoms of ARI, only additional symptoms, maternal education, maternal marital status and facility delivery remained associated with appropriate care-seeking in the adjusted analysis (Table 4). Children who had fever and/or diarrhoea in addition to ARI had double the odds of appropriate care-seeking (AOR = 2.11 [1.06–4.17]). There was strong evidence that mothers with secondary education and above were up to five times more likely to seek appropriate care for children with ARI compared with mothers with no education (AOR = 4.07 [1.62–10.23]). Women who had delivered in a health facility had higher odds of appropriate care-seeking compared with those who did not (AOR = 1.79 [1.04–2.42]).

**Table 4:** Logistic regression analysis of factors associated with appropriate care-seeking behaviour for children under five with ARI symptoms (N = 765).

Characteristics	N children	Appropriate care-seeking	Crude OR (95% CI)	p-value	Adjusted OR (95% CI)	p-value
**Child’s age (months)**				0.189		
0–11	198	16.5	1			
12–23	195	13.9	0.81 (0.44–1.50)			
24–35	128	12.8	0.74 (0.36–1.52)			
36–47	115	5.9	0.31 (0.12–0.79)			
48–59	129	14.0	0.83 (0.40–1.69)			
**Child’s sex**				0.584		
Male	389	14.0	1			
Female	376	12.5	0.88 (0.54–1.41)			
**At least one additional symptom (fever or diarrhoea)**				0.039		0.033
Yes	574	14.8	1.91 (1.03–3.52)		2.11 (1.06–4.17)	
No	191	8.3	1			
**Mother’s Age**				0.232		
<20	40	4.5	1			
20–34	536	14.3	3.56 (0.76–16.66)			
Above 35	189	12.1	2.93 (0.58–14.88)			
**Mother’s education**				<0.001		0.011
No education	408	7.3	1		1	
Primary	149	12.2	1.77 (0.90–3.49)		2.13 (0.98–4.64)	
Secondary and above	208	25.6	4.39 (2.47–7.79)		4.07 (1.62–10.23)	
**Father’s education**				<0.001		0.143
No education/missing	361	9.7	1		1	
Primary	116	6.5	0.64 (0.28–1.47)		0.38 (0.13–1.09)	
Secondary and above	288	20.3	2.37 (1.40–4.00)		0.56 (0.26–1.23)	
**Mother’s marital status**				0.022		0.002
Single	35	3.9	1		1	
In union	730	13.8	3.93 (1.22–12.67)		10.28 (2.40–43.98)	
**Mother’s religion**				0.907		
Christianity/others	218	13.0	1			
Islam	547	13.4	1.03 (0.59–1.80)			
**Household wealth quintile**				<0.001		0.509
Poorest	281	9.8	1		1	
Poorer	186	7.8	0.78 (0.36–1.69)		0.74 (0.31–1.77)	
Middle	148	12.9	1.36 (0.65–2.83)		0.93 (0.40–2.16)	
Richer	96	18.2	2.05 (0.96–4.35)		0.89 (0.33–2.44)	
Richest	54	36.2	5.19 (2.40–11.26)		1.67 (0.56–4.96)	
**Residence**				0.004		0.481
Urban	200	20.2	1		1	
Rural	565	10.5	0.46 (0.27–0.79)		1.27 (0.65–2.51)	
**Geopolitical zone**				0.002		0.080
North East	478	8.9	1		1	
North Central	54	28.8	4.14(1.78–9.62)		2.85 (1.16–7.00)	
North West	100	15.6	1.88 (0.58–3.79)		1.51 (0.71–3.23)	
South East	53	12.3	1.49 (0.58–3.79)		0.79 (0.25–2.47)	
South South	64	20.8	2.69 (1.31–5.54)		1.00 (0.40–2.51)	
South West	16	40.5	6.95 (1.67–28.88)		2.98 (0.83–10.72)	
**Antenatal care visits (mother’s most recent live birth)**				0.030		0.866
None	193	7.8	1		1	
At least one/missing	572	15.0	2.08 (1.07–4.03)		1.07 (0.48–2.37)	
**Facility delivery (mother’s most recent live birth)**				<0.001		0.036
No	550	9.0	1		1	
Yes	215	23.8	3.17 (1.97–5.11)		1.79 (1.04–2.42)	
**Distance to health facility as barrier to accessing care (mother’s self-report)**				0.034		0.498
Important barrier	305	9.2	1		1	
Not important barrier	460	15.6	1.83 (1.05–3.20)		1.25 (0.65–2.42)	
**Mother experienced previous child death**				0.056		0.216
No	502	15.1	1.00		1	
Yes	263	9.6	0.59 (0.35–1.01)		0.69 (0.39–1.24)	

Among children with diarrhea, child age, additional symptoms, religion, antenatal attendance and facility delivery were associated with care-seeking from a formal provider in the multivariable model (Table 5). The strongest risk factor was administration of ORS and zinc at home, for which children had 4.5 [3.71–5.46] times higher odds of being taken to a formal provider for care. Having fever or ARI alongside diarrhoea was positively associated with seeking care in an appropriate place (AOR = 1.45 [1.22–1.73]). Mothers who received antenatal care for the most recent birth had increased odds of appropriate care-seeking. There was no evidence of an association between maternal age, paternal education, wealth or distance to health facility and care-seeking in an appropriate place, in the adjusted model.

**Table 5:** Logistic regression analysis of factors associated with seeking care in appropriate place for children under five with diarrhoea (N = 3,956).

Characteristics	N children	Formal place	Crude OR (95% CI)	p-value	Adjusted OR (95% CI)	P-value
**Child’s age (months)**				0.004		0.027
0–11	965	25.3	1		1	
12–23	1,229	31.0	1.32 (1.08–1.63)		1.23 (0.98–1.55)	
24–35	800	24.2	0.94 (0.72–1.22)		0.87 (0.66–1.14)	
36–47	560	26.1	1.04 (0.80–1.36)		1.04 (0.79–1.38)	
48–59	402	23.5	0.91 (0.66–1.25)		0.82 (0.82–1.13)	
**Child’s sex**				0.501		
Male	2,015	27.3	1			
Female	1,941	26.2	0.95 (0.80–1.11)			
**At least one additional symptom (fever or ARI)**				<0.001		<0.001
No	1,754	23.4	1			
Yes	2,202	29.5	1.37 (1.15–1.64)		1.45 (1.22–1.73)	
**Mother’s Age**				0.142		0.436
<20	246	21.2	1		1	
20–34	2,805	27.5	1.41 (1.00–1.98)		1.29 (0.87–1.93)	
Above 35	905	26.2	1.32 (0.91–1.92)		1.26 (0.81–1.95)	
**Mother’s education**				0.384		
No education	2,248	25.7	1			
Primary	614	28.3	1.14 (0.89–1.46)			
Secondary and above	1,094	28.3	1.14 (0.92–1.42)			
**Father’s education**						
No education	1,675	24.5	1	0.017	1	0.353
Primary	481	26.9	1.13 (0.87–1.47)		1.03 (0.77–1.38)	
Secondary and above	1,574	30.4	1.34 (1.09–1.65)		1.13 (0.89–1.43)	
Missing	226	19.3	0.73 (0.48–1.13)		0.74 (0.47–1.18)	
**Mother’s marital status**				0.338		
Single	157	22.5	1			
In union	3,799	27.0	1.27 (0.78–2.10)			
**Mother’s religion**				0.100		0.249
Christianity/others	947	24.1	1		1	
Islam	3,009	27.5	1.20 (0.97–1.48)		1.21 (0.88–1.67)	
**Household wealth quintile**				0.011		0.088
Poorest	1,303	26.7	1		1	
Poorer	1,031	24.3	0.88 (0.68–1.14)		0.81 (0.62–1.06)	
Middle	776	25.3	0.93 (0.71–1.23)		0.70 (0.52–0.93)	
Richer	552	28.3	1.08 (0.82–1.44)		0.67 (0.49–0.93)	
Richest	294	36.0	1.55 (1.14–2.10)		0.81 (0.54–1.21)	
**Residence**				0.767		
Urban	1,098	27.2	1			
Rural	2,858	26.6	0.97 (0.78–1.20)			
**Geopolitical zone**				0.001		0.009
North East	1,580	25.7	1		1	
North Central	554	19.0	0.68 (0.48–0.95)		0.71 (0.51–1.00)	
North West	1,234	29.2	1.19 (0.89–1.61)		1.02 (0.75–1.39)	
South East	232	28.8	1.17 (0.79–1.73)		1.15 (0.71–1.86)	
South South	162	21.9	0.81 (0.51–1.28)		0.83 (0.50–1.39)	
South West	194	35.6	1.60 (1.08–2.37)		1.39 (0.93–2.09)	
**Antenatal care visits (mother’s most recent live birth)**				<0.001		0.076
None	1,048	19.9	1		1	
At least one/missing	2,908	29.3	1.67 (1.34–2.07)		1.24 (0.97–1.58)	
**Facility delivery (mother’s most recent live birth)**				<0.001		0.007
No	2,778	24.5	1		1	
Yes	1,178	32.4	1.48 (1.23–1.78)		1.38 (1.09–1.73)	
**Distance to health facility as barrier to accessing care (mother’s self-report)**				0.002		0.108
Important barrier	1,320	22.2	1		1	
Not important barrier	2,636	28.8	1.42 (1.14–1.76)		1.23 (0.96–1.57)	
**Previous child death**				0.696		
No	2,457	26.5	1			
Yes	1,499	27.2	1.03 (0.87–1.23)			
**ORS + zinc given**				<0.001		<0.001
No	3,094	18.8	1		1	
Yes	862	52.8	4.83 (3.99–5.85)		4.50 (3.71–5.46)	

## Discussion

Our results reveal that appropriate care-seeking is rare for three common illnesses among children under five, across socioeconomic characteristics, in Nigeria. Although at least two-thirds of caregivers sought care for their ill child, only 13–15% sought care in a timely way from a formal provider for fever and ARI, and only 27% sought care from a formal provider for children with diarrhoea. A substantial proportion of caregivers who sought care did so after the day following symptom onset or from informal providers, preventing children from receiving the correct treatment in a timely way. Moreover, no care was sought for at least one quarter of children with fever and ARI, and one third of children with diarrhoea, highlighting a need to encourage parents to seek care, as well as improve where and when they seek care.

The high burden of morbidity and mortality among under-fives in Nigeria is illustrated by one in four children reportedly having fever in the two weeks preceding the survey (in line with other studies [22]), and one in three mothers having previously lost a child in our study. Less than half of the children with fever and ARI in our study had care sought for them on the same day or day after the onset of illness. Timely care-seeking is important to minimise complications from illness, including child death, with improved survival and prognosis for good child health if care is sought in a timely manner, before complications arise [13, 23, 24]. A hospital-based study in South-West Nigeria reported an average of at least 3 days before seeking care for children with pneumonia [25]. Surveys in several Nigerian regions have identified factors contributing to delay in care-seeking such as difficulties recognising signs and symptoms, attributing illnesses to spiritual causes, waiting to see if symptoms improve, and preference of self-medication (left-over medications from previous similar illnesses) or traditional medicines [16, 26, 27].

In addition to delays in care-seeking, around half of parents who sought care for their children did so from informal places, of which the majority were informal drug shops. Studies across Latin America, Africa and Asia have described a strong preference for pharmacies and drug stores as first point of call for care-seeking for pneumonia, diarrhoea and malaria [28–30]. In Nigeria, drug shops are abundant in communities, easily accessible, available and affordable [16], explaining their popularity. However, most drug shop providers are not formally trained, licensed to prescribe medications such as antibiotics and antimalarials, or equipped to manage complications.

Although 98% of caregivers of children with fever sought care within 48 hours of onset in a community-based study in Imo state, South-East Nigeria, only 19% sought care from formal health facilities [31]. Previous research suggested that there is increased likelihood of receiving appropriate care if formal health facilities are visited [13, 23, 24]. Moreover, seeking care from informal providers may indirectly contribute to delays in receiving the correct treatment, if parents resort to formal health facilities only after informal providers’ regimens have failed.

Risk factors for appropriate care-seeking differed between the three childhood illnesses. Maternal education had positive impact on care-seeking behaviours for ARI, but not fever or diarrhoea. Mothers, being the primary caretakers, are responsible for spotting signs of ill health in their children [32] and educated mothers are more likely to have knowledge of aetiology of the diseases, recognise symptoms and danger signs; these are important to prevent delays in deciding to seek care [33]. Educated mothers are also more likely to have more resources (including financial), increased agency for self-decision and therefore can seek care without waiting for their husband’s approval [16]. The positive effect of maternal education on child health has been extensively documented [29, 34, 35]. Father’s education was associated with appropriate care-seeking for children with fever, which may similarly operate through improved knowledge or availability of resources.

Similar to other studies [8, 36], having additional symptoms was associated with increased appropriate care-seeking for ARI and diarrhoea, but not fever. Our data did not allow us to determine reasons for this, however we hypothesise that the lack of association for fever may be due to fever being considered the more severe symptom, leading parents to react to fever in the same way whether or not other symptoms are present. Antenatal attendance and facility delivery for the mother’s most recent birth, two proxies for access to care, were positively associated with appropriate care-seeking for fever and diarrhoea; however, for ARI, no association with previous antenatal attendance was found (perhaps due to the smaller sample). Having administered ORS and zinc at home was the strongest predictor of care-seeking from a formal provider for diarrhoea, indicating that caregivers who follow treatment recommendations are also more likely to turn to formal providers for care.

Islamic religion was associated with appropriate care-seeking for children with fever and diarrhoea. Although Christianity is generally believed to encourage Western medicine [16], Muslim women have been found to be very willing to use formal health services in Nigeria [37]. We were unable to explain this difference in care-seeking practices based on the data collected in the DHS. Rural/urban residence was not associated with appropriate care-seeking for any illness and wealth was only associated with increased odds of appropriate care-seeking for fever. The lack of association could be due to relatively well distributed PHCs throughout Nigeria [38].

Contrary to our hypothesis, mothers who had experienced a previous child death were less likely to seek appropriate care for their children with fever (no association for ARI or diarrhoea). Although we had hypothesised that mothers with a previous child death may encourage earlier care seeking with subsequent childhood illnesses [39], enduring barriers to accessing care contributing to a previous child death may continue to prevent appropriate care-seeking. Moreover, poor perceived quality of care potentially contributing to the previous death may result in a loss of trust in health facilities.

### Strengths and limitations

The main strength of our study is that, building on the existing literature, we jointly examined timeliness and source of care in assessing the appropriateness of care-seeking for three of the most common childhood illnesses in Nigeria. We used the most recent 2018 Demographic and Health Survey to produce a nationally representative study of prevalence and determinants of appropriate care-seeking for the most common childhood illnesses in Nigeria, which continue to be the leading causes of deaths under five.

However, there are limitations worth noting. Our study relied on maternal report of childhood illnesses and care-seeking, which may be biased. Although there is likely to be only limited recall bias within the two-week recall period, social desirability bias may have led some caregivers who did not seek care, sought care late, or from informal providers to report incorrectly, leading us to overestimate appropriate care-seeking. However, since the overall percentage of appropriate care-seeking in this study remains low, any overestimate would have little effect on the interpretation of our findings. Moreover, maternal report has been shown to be valid for care-seeking for childhood illness in Zambia [40]. Another limitation to this study was the small sample size for children with symptoms of ARI, allowing limited statistical power to detect associations. Moreover, the DHS do not collect information on hospitalisation or complications, we were therefore unable to examine the association between care-seeking behaviour and child health outcomes.

### Recommendation for programmes and research

Improvements in timeliness and source of care-seeking are urgently needed to prevent severe child illnesses and deaths in Nigeria. Widespread education campaigns are needed to increase awareness of the importance of timely care-seeking from formal facilities for childhood illnesses, across all socio-economic groups. Routine contacts including immunization, antenatal and postnatal appointments offer opportunities for health providers to remind parents when and where to seek care. Use of antenatal care and facility deliveries is much lower in Nigeria than other sub-Saharan African countries, highlighting a broader need to improve perceptions of and willingness to use the healthcare system throughout the life course. Quality improvement interventions and health system strengthening must go hand in hand with education efforts to ensure that children brought to facilities receive high-quality care, and enable a reduction in child mortality [41, 42].

Qualitative studies are needed to better understand reasons for care-seeking practices and variation across childhood diseases, and design communication campaigns. The impact of novel interventions on care-seeking behaviours should be assessed to identify successful programmes. The Covid-19 pandemic caused significant disruptions to routine health care provision globally, including sub-Saharan Africa [43, 44], with indirect impacts on child health in Africa [43]. Caregivers may be more reluctant to seek care in health facilities during the Covid-19 pandemic [44], exacerbating inadequate care-seeking practices. Epidemics such as Covid-19 and Ebola further emphasize the need for clear communication to parents on when to bring their children to hospital.

## Conclusion

This study demonstrates that appropriate care-seeking is low among caregivers of children with fever, diarrhoea and ARI symptoms in Nigeria. There is an urgent need for a national, multi-pronged communication programme to improve care-seeking for childhood illnesses, in order to reduce the unacceptably high burden of child deaths in Nigeria.
